# Scaling up Family Medicine in Uganda

**DOI:** 10.4102/phcfm.v6i1.664

**Published:** 2014-10-23

**Authors:** Innocent K. Besigye, Jane F. Namatovu

**Affiliations:** 1 Department of Family Medicine, College of Health Sciences, Makerere University, Uganda

## Abstract

It is evident that politicians, health managers and academics are realising the potential contribution of Family Medicine to health systems in sub-Saharan Africa. The challenge is in training institutions to recruit and train enough Family Physicians in order to meet expectations. The 3rd Family Medicine Conference in Uganda, held in October 2013, explored innovative ways of scaling up Family Medicine training and practice in Uganda.

## Introduction

Family Medicine is one of sub-Saharan Africa’s rapidly-developing medical disciplines.^1,2^ It is a recognised specialty in several African countries such as Botswana, South Africa, Ghana and Kenya.^3,4^ At the same time, a lot is expected of this specialty, especially as politicians, health managers and academics continue to realise its potential contribution to health systems in sub-Saharan Africa.

## Relevance of primary healthcare and Family Medicine

Since the publication of the World Health Report 2008, which emphasised that primary healthcare (PHC) is needed now more than ever before,^5^ strategies have been proposed regarding how to achieve this vision effectively. Family Medicine has been documented as the best way to achieve this vision;^6^ a view which is held by many. Family Medicine provides person-centered, as well as family- and community-oriented care. This puts Family Medicine in a position to make a significant contribution toward strengthening PHC and/or the district health system. Therefore, all countries have been urged to train enough family physicians to provide clinical care and leadership to PHC teams.^7^ The challenge is in how to recruit and train enough family physicians to meet the demand, given some of the unrealistic expectations that exist toward the discipline.

## Scaling up Family Medicine in Uganda

**FIGURE 1:**
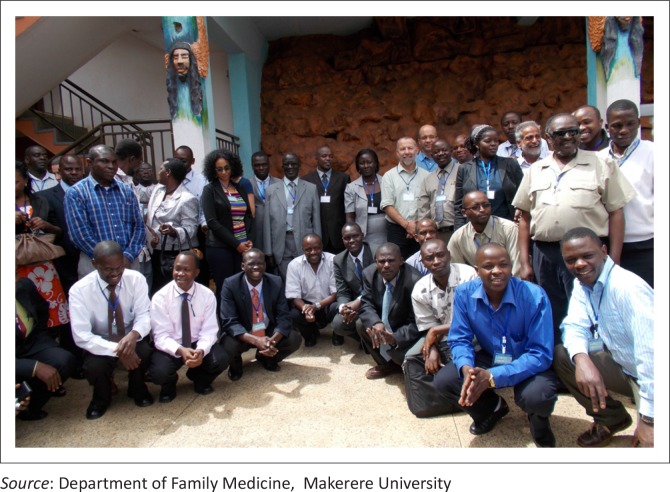
Group photograph of the conference participants.

At the 5th Primafamed workshop in Zimbabwe, participants agreed to do all that is possible to scale up Family Medicine in sub-Saharan Africa, by drawing on success stories on the African continent.^8^ The idea of scaling up Family Medicine in Uganda was first conceived in 2005 at a national dialogue held in Kampala on the future of Family Medicine.^9^ At this dialogue, a projection was agreed upon to train 400 family physicians in order to meet the set target of one family physician per 75 000 Ugandans. Since then, no significant progress has been made because only 20 family physicians have been trained. In October 2013, representatives from universities all over East Africa, various countries from all over the world (the United States of America, Canada, South Africa, Sweden and the United Kingdom), together with Ugandan Family Physicians, converged in Kampala for the 3rd National Family Medicine Conference with the theme of scaling up Family Medicine in Uganda. A group photograph showing the participants can be seen in Figure 1. They revisited the idea and discussed in detail the various ways in which Family Medicine can be scaled up in Uganda. A projection of 600 family physicians to be trained in the next 10 years was agreed upon in order to achieve a ratio of one family physician per 50 000 Ugandans.

The Ugandan health system is composed of both public and private sectors. The public sector includes all facilities owned by the government of Uganda, whilst the private sector consists of private facilities: not for profit facilities, private health practitioners and traditional and complementary medicine practitioners. The health services are structured into National Referral Hospitals (NRHs), Regional Referral Hospitals (RRHs), general and/or district hospitals and Health Centres IV to I, with health centre I being a Village Health Team (VHT).

The provision of health services in Uganda is decentralised, with districts and health centres playing a key role in the delivery and management of health services at these levels. In Africa, it has been accepted that the clinical practice of Family Medicine is integral to the district health system and includes care at the community, health centre and hospital levels. Therefore, an adequate number of family physicians is needed in order for the decentralised health system to perform effectively. Currently, there are three Family Medicine training programmes in Uganda: at Makerere University (MAK), Mbarara University of Science and Technology (MUST) and International Health Sciences University (IHSU). MAK and MUST are public universities, whilst IHSU is a private university. There are 18 Family Medicine trainees in Uganda at present, with MAK having 10, MUST one and IHSU seven. At this rate, it is not possible to train enough family physicians to meet the existing need.

In Uganda, Family Medicine was recognised immediately at its inception in 1989. Family physicians are registered as specialists by the Uganda Medical and Dental Practitioners’ Council – the licensing and/or regulatory body for health workers in Uganda. The Ministry of Health has employment positions for family physicians in NRHs, RRHs and general hospitals, where they perform various roles as hospital directors and heads of Community Health departments, as well as clinicians caring for both in- and out-patients. Some head district health systems as District Health Officers, where they provide leadership to district health teams.

## Challenges

Several issues still surround the scaling up of Family Medicine in Uganda. There is no regular and continuous funding for Family Medicine training. This, coupled with a lack of training posts, makes it difficult to recruit adequate numbers of trainees. Family physicians employed by the Ministry of Health are recruited and promoted as public health specialists. It is only those in academic medicine and non-governmental organisations that are employed and promoted as family physicians. This downplays the contribution and visibility of Family Medicine to healthcare delivery as these five-star doctors are only known as good doctors, not family physicians.

## Recommendations and conclusion

It was resolved during the conference that it is not acceptable for Uganda to keep training family physicians at this slow pace. The following suggestions were agreed upon by all the participants as a way of scaling up Family Medicine in Uganda:

- There is a need for a paradigm shift from training family physicians in tertiary and/or teaching hospitals to training them in community health centres (model family practices).
- Academic departments of Family Medicine and the Association of Family Physicians of Uganda should work together with the Ministry of Health to include Family Medicine in the Ugandan Health Sector Strategic and Investment Plan.
- Family physicians should increase their visibility through working hard and being good role models.
- Academic departments of Family Medicine and the association of the Family Physicians of Uganda should work together with the Ministry of Health to attract Family Medicine trainees, for example, through the allocation of scholarships ring-fenced for Family Medicine.
- Model family practices should be set up to demonstrate Family Medicine practice in the Ugandan context. These model practices can be public or private but with the aim of demonstrating the value of Family Medicine in the Ugandan context.
- A campaign should be instituted in order to sensitise medical officers and/or doctors about Family Medicine.
- Family physicians should be employed and promoted in their own specialty as opposed to the current situation where they are employed and promoted as public health specialists.
- Family physicians should also be promoted to the highest rank of senior consultant as other specialties. Currently, they can only be promoted to the rank of consultant.
- There are many successful family physicians in Uganda, both in the private and public sector. Their stories should be documented and published to demonstrate that Family Medicine can be a satisfying career.
- Partnerships should be formed at national and regional level with governments and other organisations such as the East African community to promote Family Medicine.

The above suggestions may be limited to, but not unique to, the Ugandan situation. As many countries, especially those in sub-Saharan Africa, struggle to scale up Family Medicine, we need to share our experiences, strategic efforts, successes and failure attempts so that we learn as much as possible from each other.
